# Sleep Debt and Postprandial Metabolic Function in Subclinical Cardiometabolic Pathophysiology

**DOI:** 10.4172/2161-1017.1000e107

**Published:** 2012-06-17

**Authors:** Barry E. Hurwitz

**Affiliations:** Behavioral Medicine Research Center, University of Miami, Miami, USA

Compared with 4 decades ago, more than twice the adults (37%) are voluntarily restricting sleep from 8.5 hours to less than 7 hours per night [1–3]. Sleep curtailment has consequences that involve not just diminished daytime performance and enhanced sleepiness and fatigue [4], but may also facilitate disease mechanisms, manifesting in a high comorbidity of sleep dysfunction and subclinical cardiometabolic pathophysiology [5,6]. Indeed, epidemiological studies have linked chronic shortened and/or poor sleep with obesity [7,8], and then also with type2diabetes disease and cardiovascular [9–12]. Despite the attention of the health profession, the media, the public and mass educational campaigns about the benefits of healthier diets and increased physical activity, the prevalence of obesity in the United States has more than doubled over the past four decades [13,14]. This increasing prevalence constitutes a major public health challenge. In persons with obesity, cardiometabolic pathogenesis occurs prematurely, and is accelerated by factors that are likely to be a combination of the established risk factors, as well as underlying insulin and glucose metabolic dysfunction associated with prediabetes and metabolic syndrome (MetS) [15]. The impact of sleep curtailment on cardiometabolic processes has not been examined as a function of obesity, prediabetes, or MetS status. Sleep loss could contribute to the development of prediabetes and MetS by deleteriously affecting glycemic regulation and/or by influencing hunger, appetite, and feeding behavior and ultimately result in weight gain and obesity [5]. Evidence suggests that central obesity and insulin resistance may be central mediating pathways by which numerous biobehavioral factors, including sleep debt, drive subclinical cardiometabolic pathophysiology [16].

In our modern world, the frequency of meals and snacks is such that each day people are in an absorptive state for most of the hours that they are awake. A study of daily glycemic excursions in persons with type 2 diabetes underscores the potential contribution of postprandial assessment to the understanding of subclinical cardiometabolic pathophysiology [17]. In those with good glycemic control (i.e., HbA1c < 7%), broad glycemic variations throughout the day were observed, wherein up to 80% of these subjects displayed postprandial glucose values above 160mg/dl. Moreover, the correlations of postprandial glycemia with fasting glucose and HbA1c values were only moderate. These findings suggest that measures of postprandial glycemia may provide more information than is provided by each of the standard alternative glycemia measures. There is also considerable epidemiological evidence indicating that the postprandial hyperglycemia, even in the absence of fasting hyperglycemia, is substantially predictive of increased CHD risk [18–22].

Recent evidence suggests that exposure of the circulatory system to repeated meal-induced waves of metabolic substances and their secondary products over the course of the day may accentuate vascular endothelial pathophysiology [23]. The pathway by which a carbohydrate meal or a high fat meal facilitates these deleterious processes is not yet fully understood but may include a mechanism by which postprandial glycemia, insulinemia, free fatty acids and/or triglyceride rich lipids drive the production of proinflammatory cytokine release and oxygen radical species [24,25]. Research is needed to study postprandial processes longitudinally across days, weeks or longer, wherein interactive and compensatory metabolic processes may be more readily observed.

Animal studies have indicated that food deprivation results in a sleep duration decrement [26], whereas total sleep deprivation stimulates excessive feeding [27]. Most sleep deprivation research has focused on its detrimental effect on CNS functioning to the exclusion of peripheral systemic function [28]. However, the notion that sleep loss is linked with body weight, feeding behavior and comorbid prediabetes and MetS conditions has been supported by recent studies demonstrating that acute sleep curtailment in healthy individuals can result in severe metabolic and hormonal consequences and changes in hunger and appetite perception [29]. These studies have typically employed procedures to restrict sleep duration by about 4 hours per night for 2 to 6 consecutive nights [30]. Longer durations of curtailment typically result in more substantive effects, but the effects can be seen even with only 2 nights of sleep curtailment [28]. This relatively immature literature has employed simple within-subject designs, with small sample sizes usually restricted to men, and has not evaluated sleep curtailment in the context of cardiometabolic risk. Another relevant issue that has not been addressed to this point is the role of sleep debt chronicity and whether prolonged sleep debt manifests in a persistently exacerbated metabolic derangement or a compensatory metabolic normalization. In sum, although it appears that acute sleep loss impacts meal-related metabolic function deleteriously, further investigation is needed on the mediating influence of sleep debt and postprandial metabolic dysregulation on weight gain, obesity and subclinical cardiometabolic pathophysiology.
